# Defending access to reproductive health information

**DOI:** 10.1093/hropen/hoaf016

**Published:** 2025-03-25

**Authors:** Maria Ekstrand Ragnar, Karin Hammarberg, Alexandra Carvalho, Ilse Delbaere, Anita Fincham, Joyce Harper, Münevver Serdarogullari, Mara Simopoulou, Christiana Antoniadou Stylianou, Randi Sylvest, Bola Grace, International Reproductive Health Education Collaboration (IRHEC), Maria Ekstrand Ragnar, Karin Hammarberg, Alexandra Carvalho, Ilse Delbaere, Anita Fincham, Joyce Harper, Münevver Serdarogullari, Mara Simopoulou, Christiana Antoniadou Stylianou, Randi Sylvest, Bola Grace

**Affiliations:** Department of Health Sciences, Faculty of Medicine, Lund University, Lund, Sweden; School of Public Health and Preventive Medicine, Monash University, Melbourne, Australia; Reproductive Medicine Unit, Gynecology, Obstetrics, Reproduction and Neonatology Department, Unidade Local de Saúde de Coimbra, Coimbra, Portugal; Health Promotion, VIVES University of Applied Science, Kortrijk, Belgium; Fertility Europe, Evere, Belgium; UCL EGA Institute for Women’s Health, University College London, London, UK; Department of Histology and Embryology, Faculty of Medicine, Cyprus International University, Northern Cyprus, Turkey; Department of Physiology, Medical School, National and Kapodistrian University of Athens, Athens, Greece; Department of Physiology, Medical School, National and Kapodistrian University of Athens, Athens, Greece; The Fertility Clinic, Department of Gynecology, Fertility and Obstetrics, Copenhagen University Hospital - Rigshospitalet, Copenhagen, Denmark; UCL EGA Institute for Women’s Health, University College London, London, UK

Sir,

Recent events in the USA serve as a stark reminder of the precarious nature of access to evidence-based information on sexual and reproductive health and rights (SRHR). Beginning just hours after President Trump assumed office, government-led efforts have rapidly and systematically dismantled vital sources of information. For example:

- The new administration removed the entire government-run website ReproductiveRights.gov. This website provided important resources and information on reproductive health care and abortion access.
- References to abortion-related policies that protect care, specifically those that safeguard access to care, have been completely erased from The Department of Health and Human Services website.
- Contraception guidelines, LGBTQ+-specific HIV testing information, and entire sets of research data from the Centers for Disease Control and Prevention website have been removed.
- All mentions of Health Insurance Portability and Accountability Act (HIPAA) protections for reproductive health care have been removed from the website, which will make it more difficult for patients to understand and advocate for their privacy rights (Centre for Reproductive Rights, 2025).

The systematic erasure of reproductive health information and research data is not just a policy shift—it is a direct assault on human rights and public health. The attack on the health and research community (The Lancet, 2025) with the banning of words (van Daal *et al.*, 2025) has serious consequences. These actions do not only restrict access to critical knowledge-enhancing information but also have the potential to exacerbate health inequalities. In the short term, they can reduce access to healthcare, information, and resources, especially for disadvantaged groups. In the longer term, they deepen systemic disparities, making it even harder for these groups to achieve good health outcomes.

Funding and research on reproductive health are already being impacted (American Society for Reproductive Medicine, 2025). When evidence-based sources of information disappear, they are easily replaced with misinformation, fearmongering, and a potential infodemic (Grace *et al.*, 2023). This disproportionately harms those already facing barriers to good healthcare: women, young people, low-income communities, LGBTQI+ individuals, and racial minorities. The potential consequences are dire, including increased rates of unintended pregnancies, unsafe abortions, and the spread of preventable diseases, as well as limited access to reproductive options and treatments (Harman, 2025). The suppression of accurate, science-based information leaves individuals unable to make informed choices about their bodies and health, undermining autonomy and well-being on a global scale.

These alarming developments underscore the urgency of global efforts to safeguard access to independent, evidence-based reproductive health education. The International Reproductive Health Education Collaboration (IRHEC), a multidisciplinary global initiative within ESHRE, is dedicated to improving reproductive health education (IRHEC, 2025). We strongly oppose the current censorship of crucial reproductive health information and reaffirm our commitment to ensuring that various stakeholders including individuals, healthcare professionals, educators, and policymakers continue to have access to scientifically accurate SRHR information and educational resources. In line with the World Health Organization, our goal is to bridge information gaps and reinforce reproductive health as an essential human right (WHO, 2025).

We must collectively raise our voices against these restrictions and censorship, recognizing the dangerous precedent being set, even in long-standing democracies. The removal of critical reproductive health information from public access is not merely a domestic issue; it represents a global threat to reproductive health and rights. Through this letter, we urge the medical and scientific communities to stand firm in defending the integrity of SRHR information and advocating for transparency, education, and evidence-based policy. Failing to act risks jeopardizing decades of progress in reproductive health, endangering the lives and freedoms of millions.
